# Diagnostic guidelines for the histological particle algorithm in the periprosthetic neo-synovial tissue

**DOI:** 10.1186/s12907-018-0074-3

**Published:** 2018-08-25

**Authors:** G. Perino, S. Sunitsch, M. Huber, D. Ramirez, J. Gallo, J. Vaculova, S. Natu, J. P. Kretzer, S. Müller, P. Thomas, M. Thomsen, M. G. Krukemeyer, H. Resch, T. Hügle, W. Waldstein, F. Böettner, T. Gehrke, S. Sesselmann, W. Rüther, Z. Xia, E. Purdue, V. Krenn

**Affiliations:** 1 Department of Pathology and Laboratory Medicine, Hospital for Special Surgery, 535 E 70th Street, New York, NY 10023 USA; 2 Medizinische Universität Graz, Institut für Pathologie, Graz, Austria; 3 Pathologisch-bakteriologisches Institut, Otto Wagner Spital, Wien, Austria; 4 Department of Orthopaedics, Faculty of Medicine and Dentistry, University Hospital, Palacky University Olomouc, Olomouc, Czech Republic; 5 Department of Pathology, Fakultni Nemocnice Ostrava, Ostrava, Czech Republic; 6 Department of Pathology, University hospital of North Tees and Hartlepool NHS Foundation Trust, Stockton-on-Tees, UK; 7 Labor für Biomechanik und Implantat-Forschung, Klinik für Orthopädie und Unfallchirurgie, Universitätsklinikum Heidelberg, Heidelberg, Germany; 8 MVZ-Zentrum für Histologie, Zytologie und Molekulare Diagnostik, Trier, Germany; 9 LMU Klinik, Klinik und Poliklinik für Dermatologie und Allergologie, Munich, Germany; 10 Baden-Baden Klinik, Baden-Baden, Germany; 11 Paracelsus-Kliniken Deutschland Gmbh, Osnabrück, Germany; 12 Universitätsklinik für Unfallchirurgie und Sporttraumatologie, Salzburg, Austria; 13 Hôpital Orthopédique, Lausanne, Switzerland; 14 Medizinische Universität Wien, AKH-Wien, Universitätsklinik für Orthopädie, Wien, Austria; 15 Adult Reconstruction and Joint Replacement Division, Hospital for Special Surgery, New York, NY USA; 16 Helios Endo-Klinik, Hamburg, Germany; 17 Orthopädische Universitätsklinik Erlangen, Erlangen, Germany; 18 Klinik und Poliklinik für Orthopädie, Universitätsklinikum Hamburg-Eppendorf, Hamburg, Germany; 19 Centre for Nanohealth, Swansea University Medical School, Singleton Park, Swansea, UK; 20 Hospital for Special Surgery, Research Institute, New York, NY USA

**Keywords:** Arthroplasty, Histological particle algorithm, Periprosthetic tissue, Synovial-like interface membrane, Orthopaedic implant wear particles, Non-implant related particles, Synovial crystals, Metallic wear particles, Ceramic wear particles, Polyethylene wear particles

## Abstract

**Background:**

The identification of implant wear particles and non-implant related particles and the characterization of the inflammatory responses in the periprosthetic neo-synovial membrane, bone, and the synovial-like interface membrane (SLIM) play an important role for the evaluation of clinical outcome, correlation with radiological and implant retrieval studies, and understanding of the biological pathways contributing to implant failures in joint arthroplasty. The purpose of this study is to present a comprehensive histological particle algorithm (HPA) as a practical guide to particle identification at routine light microscopy examination.

**Methods:**

The cases used for particle analysis were selected retrospectively from the archives of two institutions and were representative of the implant wear and non-implant related particle spectrum. All particle categories were described according to their size, shape, colour and properties observed at light microscopy, under polarized light, and after histochemical stains when necessary. A unified range of particle size, defined as a measure of length only, is proposed for the wear particles with five classes for polyethylene (PE) particles and four classes for conventional and corrosion metallic particles and ceramic particles.

**Results:**

All implant wear and non-implant related particles were described and illustrated in detail by category. A particle scoring system for the periprosthetic tissue/SLIM is proposed as follows: 1) Wear particle identification at light microscopy with a two-step analysis at low (× 25, × 40, and × 100) and high magnification (× 200 and × 400); 2) Identification of the predominant wear particle type with size determination; 3) The presence of non-implant related endogenous and/or foreign particles. A guide for a comprehensive pathology report is also provided with sections for macroscopic and microscopic description, and diagnosis.

**Conclusions:**

The HPA should be considered a standard for the histological analysis of periprosthetic neo-synovial membrane, bone, and SLIM. It provides a basic, standardized tool for the identification of implant wear and non-implant related particles at routine light microscopy examination and aims at reducing intra-observer and inter-observer variability to provide a common platform for multicentric implant retrieval/radiological/histological studies and valuable data for the risk assessment of implant performance for regional and national implant registries and government agencies.

## Background

The identification of particulate wear material of orthopaedic implants and its differential diagnosis with endogenous crystalline and non-crystalline materials in the periprosthetic capsular neo-synovial membrane, bone, and the synovial-like interface membrane (SLIM) is important for the evaluation of clinical outcome, correlation with radiological and implant retrieval studies, and understanding of the biological adverse reactions associated with implant failures. The role of wear particles was first recognized by Willert and Semlitsch in 1977 in the occurrence of bone resorption leading to aseptic loosening/osteolysis, one of the most frequent causes of orthopaedic implant failure up to the present time [1].

Wear in orthopaedic implants is considered a result of removal of material by mechanical action. Implant wear particles are derived from polyethylene, metallic alloys, ceramics, implant porous coatings, and polymethyl methacrylate orthopaedic cement (PMMA). The great majority of these particles are generated by two mechanisms: 1) The two-body adhesive/abrasion wear when material is removed or displaced from the softer surface by irregularities of the harder surface; 2) The three-body abrasion wear when some form of other particles generated by materials used to fasten the implant to the bone (e.g. PMMA) or particles generated by the wear of a primary implant components which remain after the implant failure and revision (e.g. ceramic particles after a fracture of a ceramic femoral head or liner) [2]. Particulate material can also be generated by tribochemical wear (tribocorrosion) mechanism and by other modality at the head-neck tapers such mechanically assisted crevice/fretting corrosion, pitting and intergranular corrosion, and etching which depend on the material, material couple, and alloy microstructure [3, 4].

In the histological examination of capsular neo-synovial membrane, bone and SLIM, wear particles can be of any size, shape, contour, colour, and chemical composition. The differential diagnosis with non-implant related exogenous particles can be difficult or sometimes even impossible by light microscopy (e.g. presence of minute particles of surgical suture or glove powder). Large wear particles can also represent aggregates of smaller particles, especially of nano-size and also admixed with or coated by adherent organic substance, such as blood-derived products or synovial fluid proteins forming a protein corona, which can define the immunogenic properties of the particles [5–7].

In the past forty years, histological classifications of implant wear particles at conventional light microscopy examination with and without polarized light have been published and used for clinical purposes and research studies, usually modifications of the classification reported by Mirra et al. in 1976 [8]. Although the proposed classification based on a semi-quantitative scale of particle number and size is to some extent still valid, the progressive evolution of implant and non-implant related material and technological developments in microscopy optics, microscope camera and imaging, and particle analysis techniques has made the original classification and its modifications not suitable to address all the current diagnostic challenges for surgical pathologists and in need of an up-to-date classification and a comprehensive, digital photographic documentation. Moreover, the identification of wear particles and the measurement of their total burden has become more difficult or impossible to be determined with accuracy by conventional light microscopy due to the size of most of the particulate material well below the resolution limit of the optic microscope, the morphological similarities of some of the material, and the mixture of particles from different material in the cytoplasm of the macrophages.

Histological examination of the periprosthetic tissue removed during implant revisions, although still not mandatory in many countries, has been considered instrumental in the classification, cell composition/subtyping, and grading of the adverse biological reactions to implant wear particles and the identification of new types [9–15]. These reactions can potentially carry vast medical and economic consequences for public health, as exemplified by the increasing number and type of joints replaced (hip, knee, shoulder, elbow, wrist, ankle), and the projected increased number of orthopaedic implant revisions in the future [16]. A recent example has come from the re-introduction of the metal-on metal (MoM) bearing surface either in hip resurfacing arthroplasty (HRA) or total hip arthroplasty (THA) with or without cobalt-chromium (CoCr) metallic adapter sleeve (MAS) and in Non-MoM THA implants with CoCr dual modular neck (DMN) followed by the unintended occurrence of adverse local tissue reactions resulting in a higher rate of revision operations and need of long-term follow-up [17].

**Fig. 1:**
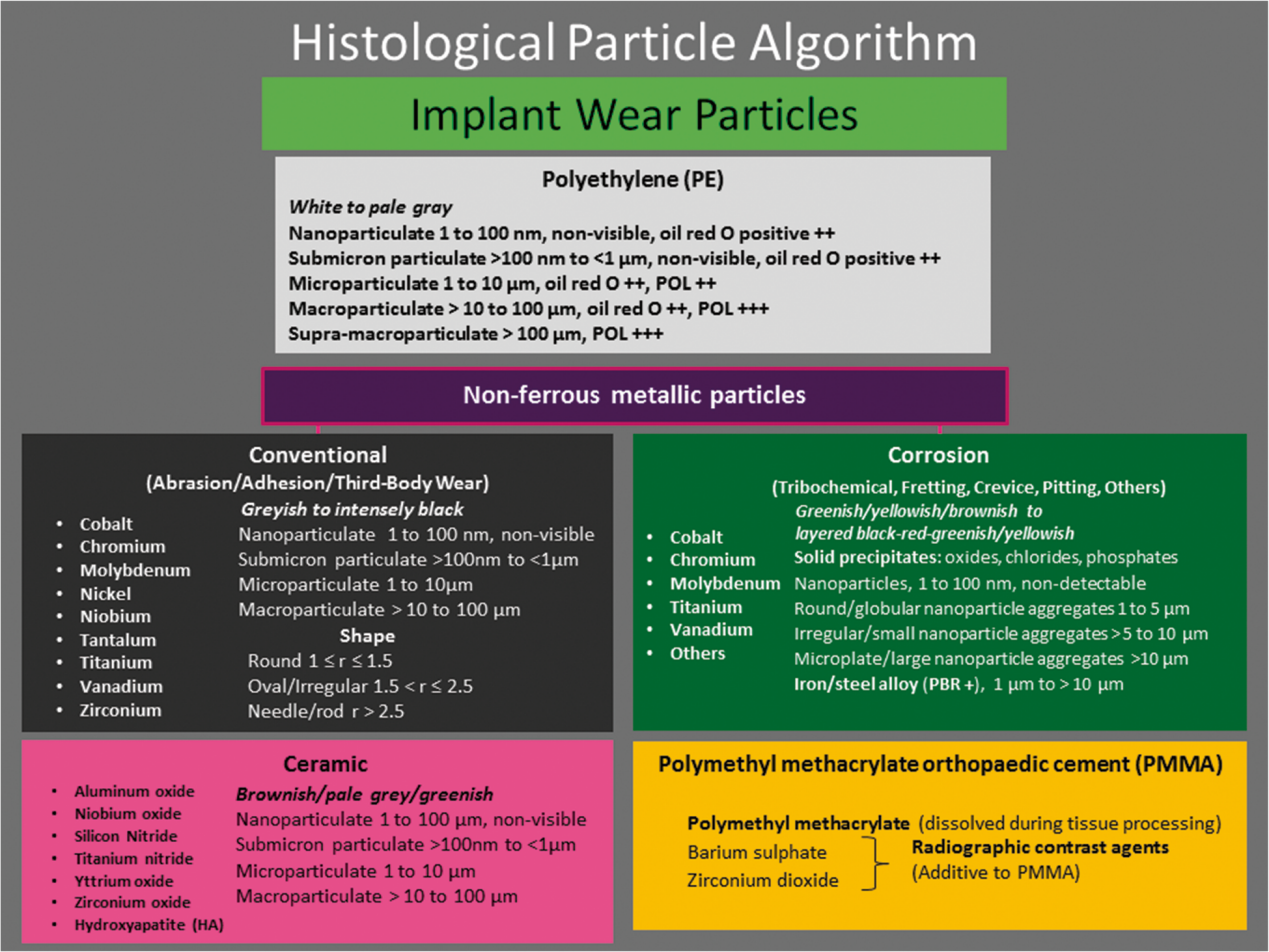
Histological particle algorithm: Implant wear particles. These particles are identified in the periprosthetic tissue/SLIM by type, color and size

**Fig. 2:**
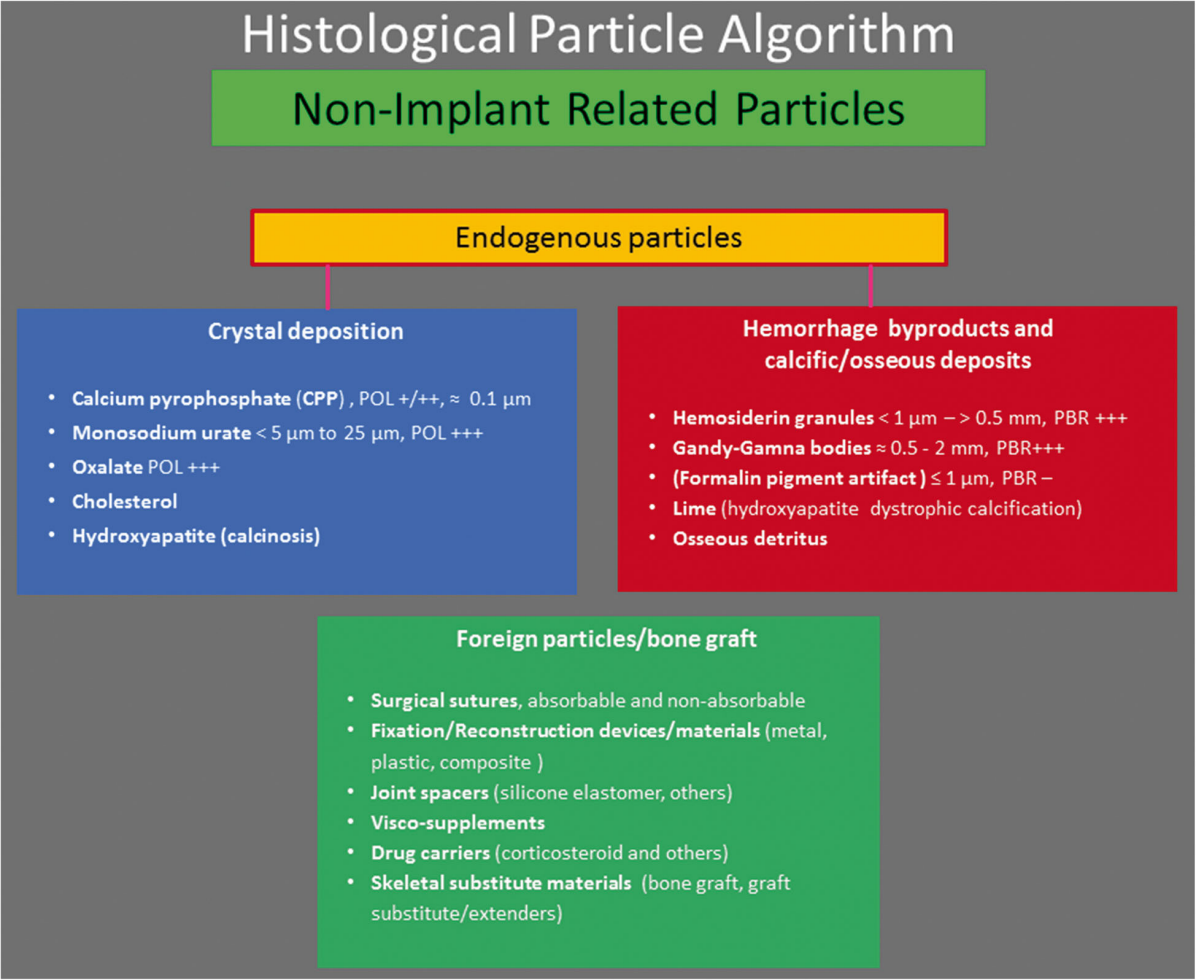
Histological particle algorithm: Non-implant related particles. These particles in the periprosthetic tissue/SLIM are of endogenous and/or foreign origin

The first analysis of the implant wear material and of the host reaction is almost always performed by conventional light microscopy on paraffin embedded tissue and usually by a general surgical pathologist. Therefore, a classification of the materials which is reproducible and accurate within the limits of the optic microscope resolution and also of limited methodological complexity is necessary for providing a standardized and comparable diagnostic tool which can be expanded further with the use of additional, sophisticated analytical techniques when necessary. A set of detailed criteria is presented with the intent of providing a useful and reproducible guide for the identification of wear particulate material (Fig. 1) and for the differential diagnosis with non-implant related endogenous materials such as crystal deposits and degradation products of organic substances, especially blood-derived, as well as foreign particles/grafts from different sources (Fig. 2). These criteria can be applied to the decisional tree of the Histological Particle Algorithm (HPA) for the correct identification of the particles which has been reported before in a condensed version [18, 19] and more recently in a manual for the histological diagnosis of pathologies associated with orthopaedic implants [20].

## Methods

The HPA is fully described and illustrated with examples of each category of implant wear particles and endogenous/foreign particles generated in different implanted joints. The cases were selected retrospectively from the pathology archives of the Hospital for Special Surgery, New York, NY, USA and the Centre with Focus on Orthopaedic Pathology, Trier, Germany. All cases were retrieved by histological diagnosis and presence of implant-wear or non-implant related material. The cases were selected on the basis of exhibiting ample evidence of presence of a specific type of particle and in some cases of a certain size range for illustrative purposes. All cases were histologically examined by three orthopaedic pathologists (GP, SS, VK) with consensus agreement on the histological diagnosis. Approval for the use of the periprosthetic tissue was obtained by the Institutional Review Board, Hospital for Special Surgery (Protocol Number 26085) and the Ethics Commission of the Medical Board of Rheinland-Pfalz; Mainz, Germany [Case Number 837.230.15 (9998)]. All particle categories were described according to their size, shape, colour, and properties observed at conventional light microscopy with or without polarized light and after histochemical stains when necessary. The criteria used in the HPA for particle identification are based in large part on the ones previously described in the scientific literature and provided in the definitions of particle size and shape section below. The particle size ranges were determined using computer-aided interactive morphometric analysis, Leica DM 2005, microsystems framework 2007 by two of the three pathologists (VK and SS) and independently verified by the third pathologist (GP) using a similar computer-aided system, Zeiss Axioskop 40, Jenoptik ProgRes microscope camera. The systems were calibrated using a standard micrometer glass slide with 1 mm horizontal scale, 100 divisions -10 μm intervals.

### Implant data

The type of prosthesis and its time of implantation were known in each case and the removed implant components available for inspection at gross examination. The material composition of the joint prostheses covered the full range of the implant material spectrum (polyethylene, metals, ceramics, silicone, hydroxyapatite, polymethyl methacrylate orthopaedic cement).

### Macroscopic examination and histological processing and staining of tissue samples

The fresh tissue collected at surgery was fixed in 10% buffered formaldehyde. Macroscopic description of the specimens was performed and depending on the wet tissue sample size/weight and/or tissue mapping locations, up to 10 tissue blocks (1–2 sections/block) were selected per case, processed and embedded according to a standard protocol, cut at 4 to 5 μm and stained with hematoxylin and eosin (H&E) and additional histochemical stains when necessary. The H&E staining and Prussian blue reaction (PBR) were carried out with a standard protocol using the Leica ST 4040 staining module; nuclear staining was undertaken with Harris hematoxylin (Harris hematoxylin, Surgipath, Richmond, IL, USA), the background staining was performed using eosin Y (Sigma-Aldrich, St. Louis, MO, USA). PBR reaction was performed manually according to the Mallory’s method and Oil-red-O staining (Sigma-Aldrich, St. Louis, MO, USA) following a standard protocol [21]. In selected cases, 0.35 μm thick sections were stained with 0.1% toluidine blue in borax buffer after fixation in 2.5% glutaraldehyde for 24 h and trimming with preservation of tissue orientation, transferred to sodium cacodylate buffer, processed through a standard cycle and embedded in epoxy resin.

We retain that sampling from multiple regions around large joints of periprosthetic tissue has value and can also provide more accurate information of the type of wear particles and their distribution as well as the quantitative and qualitative evaluation of the host cell response. This method has been previously described in detail [22] and has been used for the assessment of ALTR inflammatory infiltrate and the identification of large aggregates of corrosion particles/products [23, 24]. However, mapping for tissue sampling can be time consuming with a higher technical and professional cost to be applied routinely to all specimens and its benefits have also been recently investigated on non-MoM total hip and knee arthroplasty specimens showing comparable results with or without the use of a mapping chart [25].

### Particle analysis

We found it challenging to match the wear particle lexicon used for retrieval analysis with the categories used in the histological classification and especially for the metal-on-metal and non-metal-on metal implants with wear particles generated at taper junctions between modular components. In particular, we faced the issue of separating metallic particles generated by adhesion/abrasion, tribocorrosion and mechanically assisted fretting/crevice and other types of corrosion. Therefore, we divided the metallic wear particles into two subcategories: 1) Conventional, as the most frequently occurring particles generated by adhesion/abrasion which can be predominantly composed of Co with variable amount of Cr, Mo, and Ti elements, varying in size and color from grey to jet black and responsible for the synovial fluid staining and macroscopic appearance clinically defined as “metallosis”; 2) Corrosion, as the particles of nanosize generated at the metal-on-metal bearing surface by tribocorrosion and composed of Cr with absent or minimal CoMo component distinctive of the MoM bearing surface and the particles generated by fretting, crevice, pitting, intergranular, etching, and possibly other types of corrosion at the taper junctions and composed of variable amounts of Cr, Co, Mo, Ti, and other metals used in the alloy and which usually appear at light microscopy of greenish/yellowish colour and less frequently brownish. This matter is further complicated by the fact that both sub-categories of particles can be observed simultaneously at histological examination of periprosthetic tissue and cannot be distinguished with certainty without tissue sampling for transmission electron microscopy (TEM)/scanning electron microscopy (SEM) and subsequent particle nano-analysis. Corrosion particles have also been referred to as corrosion products or corrosion particulate material in the literature because of their composite nature of metallic particles admixed with organic substance [20, 23]. Although it would be difficult to separate them in the classification, we used the term “corrosion products” instead of “corrosion particles” in some illustrations when the particle aggregates were large and multi-layered with the addition or organic derivatives mainly from blood/synovial fluid proteins and cell debris.

Non-implant related particles present in the periprosthetic tissue are divided in two main categories: endogenous particles and foreign particles/bone grafts.

Endogenous particles are those microscopic particles in the periprosthetic tissue/SLIM which have been produced by the body, frequently secondary to metabolic diseases/disturbances or blood-derived products and degenerative processes. The majority of them are of crystalline nature and are detected either in the synovial fluid or in deposits into the articular soft tissues. For the analysis of endogenous particles, a light microscope fitted with polarized filters and a first order red compensator is sufficient [26]. Crystals are detected because of their birefringence which is evident with the use of compensated polarized light microscopy (CPLM).

Foreign particles are derived from absorbable and non-absorbable surgical sutures, fixation devices/scaffolding materials (metal, plastic, composite materials), joint spacers, skeletal substitute materials, visco-supplements, and drug carriers.

### Definitions of particle size and shape

The most difficult issue of the classification of wear and prosthetic material particles generated by orthopedic implants is to translate the size range of the particles into equivalent words to provide a practical and easily reproducible classification. This issue is not trivial because different morphological features of the particles and in particular their size and irregular surface could lead to an increase in the activation of the macrophage inflammasome with subsequent cytokine release in vivo and therefore have an important clinical significance, at least for metallic particles, as shown in an in vitro study [27]. We propose a unified range of particle size, defined as a measure of length only, which includes five classes for PE particles, and four classes for metallic particles and ceramic particles (class 1 to 4): 1) Nanoparticles (1 to 100 nm), 2) Submicron particles (> 100 nm to < 1 μm), 3) Microparticles (1 μm to 10 μm), 4) Macroparticles (> 10 μm to 100 μm), and 5) Supra-macroparticles (> 100 μm). This particle range is supported by its use for characterization of GFV UHMWPE in vitro [28] and in the attempt to predict their functional biological activity [29, 30], of metallic particles [31], and of ceramic particles [11]. It must also be taken into consideration that particles of large size, especially metallic wear debris, can be actually aggregates of particles of much smaller size with the possible addition of organic elements. For an approximate extimate of particle size at light microscopy examination, nanoparticle aggregates, submicron, and microparticles are usually present in macrophages, macro-particles in single or syncytial giant cells, and supra-macroparticles are surrounded by giant cells or free in the capsular neo-synovial stroma/SLIM. The use of a ruler reticle mounted on the eyepiece can be helpful for a more precise, still approximate measure of the particle sizes. It needs to be emphasized that light microscopy examination cannot provide a reliable estimate of particle size distribution for which other techniques such as laser diffraction, dynamic light scattering, and image analysis must be used after particle isolation or TEM/SEM for in vivo analysis of intracellular and extracellular particle content. Shape descriptors can also be used, although different terms are used for various materials [31–33]. The following sub-division is proposed for the most common wear material observed at light microscopy with the use of a polarizing filter when necessary and according to the size ranges detected for each material:

1. *Polyethylene*: nanoparticles, non-detectable (1 to 100 nm); submicron particles (> 100 nm to < 1 μm), non-detectable; microparticles (1 to 10 μm); macroparticles (> 10 μm to 100 μm); and supra-macroparticles (> 100 μm);
2. *Conventional metallic*: nanoparticles, non-detectable (1 to 100 nm); submicron particles (> 100 nm to < 1 μm), non-detectable; microparticles (1 to 10 μm); and macroparticles (> 10 μm). Their shape can be defined by the ratio (r) between the length and the width of the particles: round (1≤  *r*  ≤ 1.5), oval/irregular (1.5 < *r*  ≤ 2.5), needle/rod shaped (*r* > 2.5) [33];
3. *Corrosion metallic*: nanoparticles (1 to 100 nm), non-detectable; round/globular nanoparticle aggregates (1 to 5 μm); irregular, small nanoparticle aggregates (1 to 10 μm), usually break-down fragments of larger aggregates; microplate, large nanoparticle aggregates (> 10 μm). Nanoparticles and small aggregates are usually associated with tribocorrosion and the larger aggregates to crevice/fretting corrosion;
4. *Ceramic*: nanoparticles, non-detectable (1 to 100 nm), submicron particles (> 100 nm to < 1 μm), non-detectable, microparticles (1 to 10 μm), macroparticles (> 10 μm);
5. *PMMA*: Large particles surrounded by giant cells dissolved during tissue processing cycle and appearing as empty lacunae with small aggregates of radiographic contrast agent present; medium and small size particles engulfed in giant cells and dissolved as well; particles of radiographic contrast agent in macrophages (barium sulphate, zirconium dioxide) especially numerous in cases of three-body wear failure.

## Results

### Implant wear particles

Wear particle characterization of orthopedic implant wear is the most challenging component of the histological examination of the periprosthetic tissue and it is often time consuming, especially when multiple specimens/case and several cases have to be examined in a single session. The most frequently used bearing surface couplings are: metal-on-polyethylene (MoP), ceramic on polyethylene (CoP), metal-on-metal (MoM), and ceramic-on-ceramic (CoC); a metallic adapter sleeve, made of CoCrMo or Ti has been added to large metallic heads (≥32 mm) and of Ti to large ceramic heads (≥32 mm). PMMA cement can be present around the femoral and/or the acetabular component. Each material can be a source of particulate debris.

#### Polyethylene (PE)

**Fig. 3:**
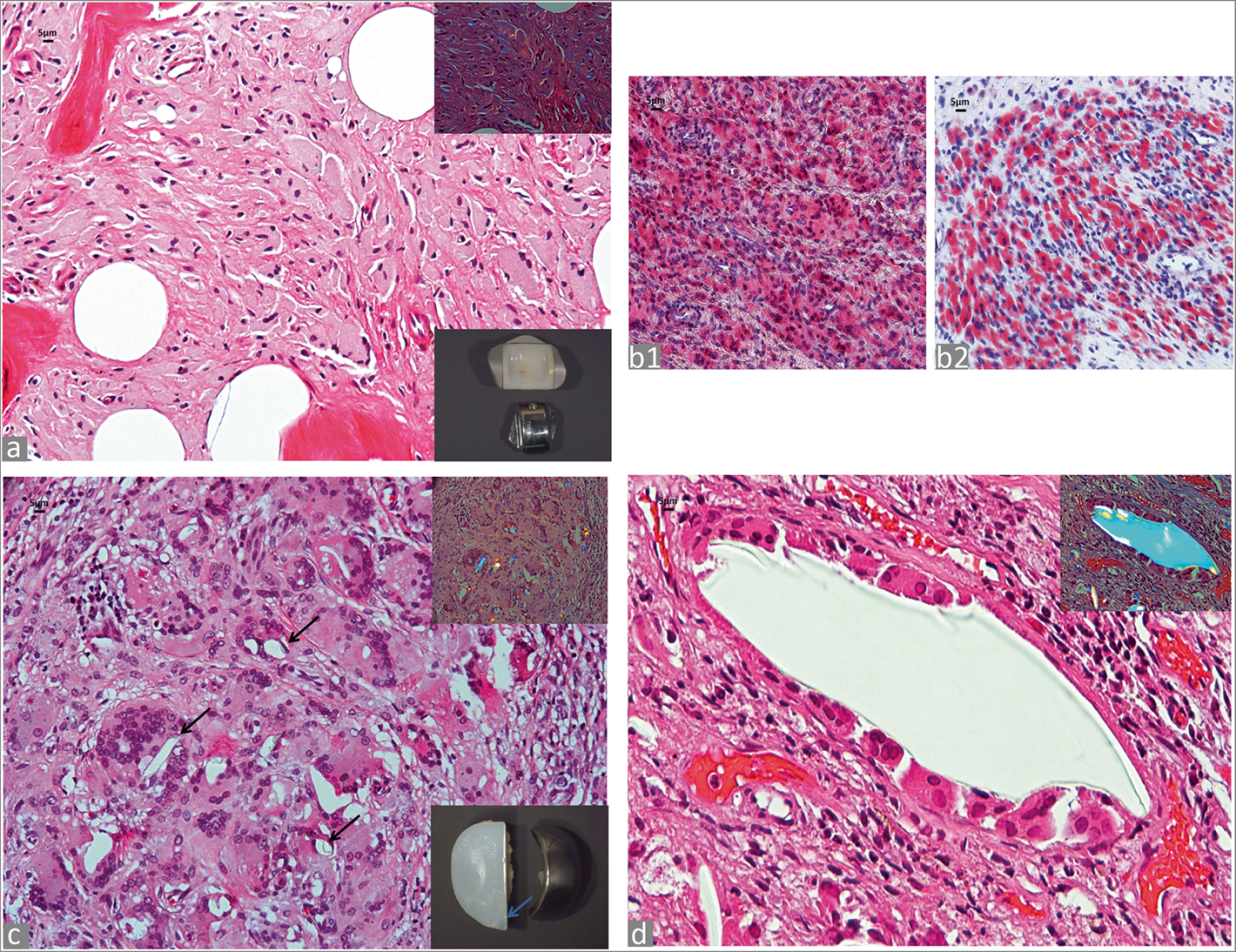
Polyethylene (PE) particles. **a** Osteolysis with diffuse macrophage infiltrate containing PE micro-particles (H&E× 200), birefringent PE micro-particles under polarized light in the upper right inset (× 400), total ankle replacement implant in the lower right inset. **b1.** Oil red O positivity for macrophage cytoplasm containing PE micro- and submicron particles in a MoP TKA implant (× 200); **b2.** Oil red O positivity for macrophage cytoplasm containing tribocorrosion metallic particles in MoM HRA implant (× 200). **c** PE macroparticles (black arrows) in multinucleated giant cells (H&E × 200), birefringent particles under polarized light in the upper right inset (× 200), left unicompartmental knee implant with large area of PE abrasion/delamination (blue arrow) in the lower right inset. **d** PE supra-macroparticle lined by multinucleated giant cells in a case of failed total elbow prosthesis (H&E × 200), birefringent PE supra-macroparticle under polarized light in inset (× 200)

The term polyethylene usually refers today to its ultra-high molecular weight type (UHMWPE) with an exceptionally high molecular mass, defined by the American Society for testing and materials as a molecular weight higher than 3.1 million g/mol [34]. Today the most common subtypes analyzed at the time of revision according to the year of implantation and type of prosthetic device are the first and second generation of highly crossed-linked polyethylene (HXLPE) subject to different regimens of radiation for sterilization and remelting/thermal heating for oxidation stability and in the future also with the addition of an anti-oxidant stabilizer, such as vitamin E [35]. The use of highly crossed-linked polyethylene varies among the different joint implants and the detailed information of the dose of radiation and of the use of remelting or thermal heating is usually not available to the pathologist at the time of the histological examination. Although the occurrence of macro- or supra-macroparticles has been typically associated with first generation non-highly cross-linked polyethylene [36], particles of large size can still be observed in implants of more recent design [37]. PE particles of variable size are detected at light microscopy examination under polarized light:

*Microparticles*:

PE microparticles are usually located in the cytoplasm of the macrophages; they measure between 1 and 10 μm and are predominantly globular, elongated, and fibrillary/needle-shaped showing variable birefringent reactivity under polarized light (Fig. 3a). Scanning electron microscopy (SEM) analysis has shown that particles of UHMWPE are round or elongated and that the former are in large majority submicron in size and therefore non-detectable at polarized light microscopy examination with predominance of the latter often around to 10 μm in length [38, 39]. They are visible at × 400 and × 200 under polarized light. The equivalent shape ratio (ESR) has been used in a recent study comparing UHMWPE to HXLPE particles in total ankle arthroplasty to characterize the particles which are classified as round (ESR < 1.5), elongated (1.5 ≤ ESR ≤ 3) and fibrillary (3 < ESR) [40]. The detectable number of particles is also dependent on the polarizer and analyzer components used for the analysis. Oil red O staining provides positive staining of cytoplasmic, micro-particulate PE (Fig. 3b1), although it is non-specific and stains also diffusely the cytoplasm of macrophages filled with metallic nanoparticles generated by tribocorrosion in MoM bearing surface implants (Fig. 3b2), most probably bound to lipids of phagosome membranes and/or lipoprotein component of the protein corona as recently described [41].

*Macroparticles*:

PE macro-particles measure between > 10 μm and < 100 μm; they are they exhibit variable shape from roundish to oval or irregular with usually smooth contour (Fig. 3c). They are detected at × 100 and × 40 magnification.

*Supra-macroparticles*:

These particles are detectable at × 25 magnification, especially under polarized light. Their shape is variable and frequently curved and they are surrounded by multinucleated giant cells or free in the stromal tissue with size ranging from 100 μm up to > 2000 μm (Fig. 3d). Particles larger than 1000 μm may be also detected at macroscopic examination.

#### Non-ferrous metallic particles

##### Conventional metallic particles

**Fig. 4:**
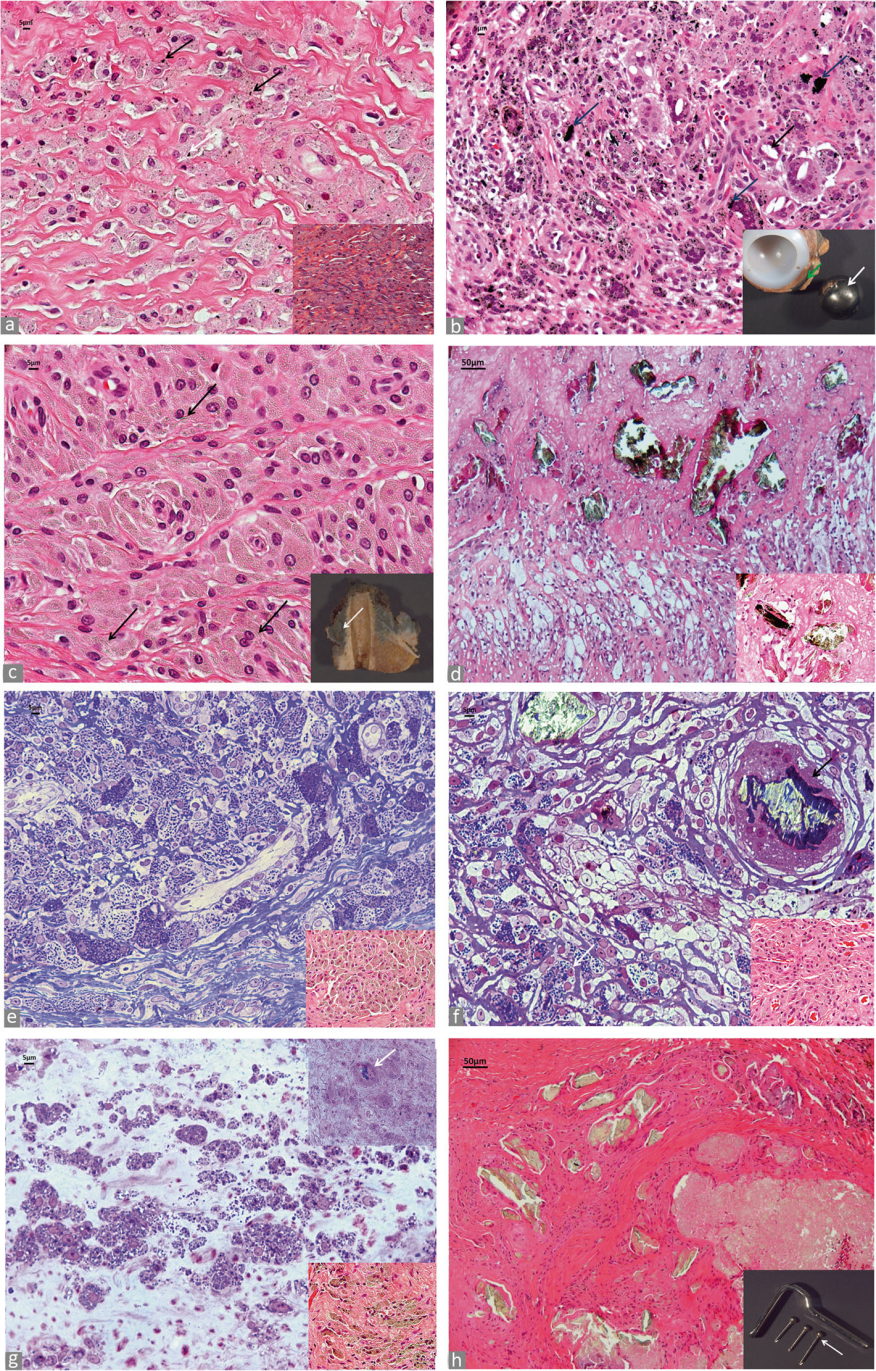
Metallic non-ferrous particles. **a** Conventional black metallic particulate debris (black arrows) in MoP implant (H&E × 400), birefringent PE debris is evident as blue, granular and needle-shaped particles under polarized light in inset (× 400). **b** Macrophagic and giant cell reaction to conventional particulate metallic debris with irregular macroparticles in multinucleated giant cells (blue arrows) with empty lacunae of methyl methacrylate orthopedic cement (black arrow) (H&E × 200), CoCr femoral head with a large band of abrasion (white arrow) secondary to edge loading due to subluxation on the distorted and cemented Ti acetabular rim (green arrow) in inset. **c** Oxidized metallic particulate debris in MoM HRA implant macrophages filled with tribocorrosion nanoparticle aggregates and rod/needle-shaped larger abrasion microparticles (black arrows) (H&E × 400); femoral head with osteolytic cavity (white arrow) and neo-synovium with charcoal-gray color, indicative of conventional metallic debris from edge loading in inset. **d** Deposits of large aggregates of greenish corrosion products in enlarged trochanteric bursa of a MoM THA implant with MAS generated at head/neck junction by mechanically assisted fretting/crevice corrosion (H&E × 100), details of particles with green (CoCrMo), red (blood-derived) and black (Ti) layers in inset (H-E × 400). **e** Macrophagic infiltrate containing predominantly tribocorrosion metallic nanoparticles in MoM HRA implant, semithin section (toluidine blue × 400); macrophagic infiltrate in inset (H&E × 400). **f** Macrophage infiltrate containing predominantly tribocorrosion metallic nanoparticles (white arrow) and microplate of corrosion product from head-neck junction in a multinucleated giant cell (black arrow) of a MoM THA implant with MAS, semithin section (toluidine blue × 400); macrophage infiltrate in inset (H&E × 400). **g** Macrophage infiltrate containing irregular metallic nanoparticle aggregates in a non-MoM THA with CoCr DMN, semithin section (toluidine blue × 400) and giant cell with a large aggregate of nanoparticles/corrosion product (white arrow), semithin section, in the upper right inset (toluidine blue × 400), macrophage infiltrate in the lower right inset (H&E × 400). **h** Microplates of corrosion particle aggregates generated at fixation device screw-plate interface (H&E × 100). Metallic plate and metallic screws with corrosion observed at the screw head/threaded body junction (white arrow) in inset

Non-ferrous metals and their alloys are used predominantly in joint endoprosthesis, where ferrous metals (i.e. steel) are used considerably less at the present time [42]. The most used non-ferrous metals in joint prosthesis are: aluminum (Al), cobalt (Co), chromium (Cr), molybdenum (Mo), nickel (Ni), niobium (Nb), tantalum (Ta), titanium (Ti), vanadium (Va), zirconium (Zr). These metals are used in various combinations and alloys. Conventional metallic wear particles are predominantly very small particles and have an average diameter ranging from 0.05 μm to < 5 μm and only occasionally are > 5 μm especially in cases of component displacement or massive abrasion by three-body wear. The shape is predominantly rod/needle-like, but can vary from round to polygonal, sharp-edged. Their colour varies from grey to jet black. Conventional metallic debris can be admixed with either polyethylene (Fig. 4a) or ceramic debris and appear in micro and macroparticulate form in cases of femoral neck impingement or marked edge loading of the acetabular rim (Fig. 4b). It is important to emphasize that CoCrMo metallic particles, either generated by tribocorrosion (corrosion particles in our classification and rich in Cr) or by abrasion/adhesion (conventional particles in our classification and rich in Cr and Co) at the MoM bearing surface of HRA and LHTHA hip implants can be also oxidized during H&E staining with Harris hematoxylin and appear pale green/yellowish or brownish at light microscopy examination, in contrast with the charcoal-grey/black appearance of the tissue at surgery and macroscopic examination (Fig. 4c). In this case, the whole blood level of Co was 91.6 μg/L and of Cr 96.5 μg/L at the time of revision. This difference in wear particles composition has been corroborated by the finding of a predominant group of amorphous particles smaller than 10 nm in the phagosomes and composed of Cr admixed with a small number of larger, high electron density needle-like particles of Cr with Co component (Cr particles density < CoCr particle density) in cases of MoM HRA when analysed by TEM and BSEM-analysis [43]. Similar findings have also been shown with particles generated by a MoM hip simulator in vitro [44]. Moreover, wear debris from implants classified under the ceramic category and composed of a wrought zirconium alloy oxidized by thermal diffusion to form a thin, surface layer of oxidized zirconium can also have a similar appearance.

##### Corrosion metallic particles

Corrosion of orthopaedic implants was considered a serious clinical concern in the late nineties, although it was believed that the adverse clinical outcomes could be minimized with attention to variables related to the selection of the materials and implant configuration, and metallurgical processing [45]. A renewed attention has been recently devoted to implant corrosion particles/products because of the adverse local tissue reactions/adverse reaction to metallic debris (ALTR/ARMD) associated with MoM HRA and MoM THA [14, 46], MoM large head THA implants with or without CoCr MAS [47, 48], non-MoM THA implants with CoCr DMN [49–51] and other MoP THA configurations [52, 53]. They are predominantly produced by tribocorrosion at the MoM bearing surface (the intended wear mode) and by mechanically assisted fretting/crevice corrosion (the unintended wear mode) and other less frequent types of corrosion at the head/neck junctions [54]. They have also been recently described in modular TKA implants [55, 56]. Direct inflammatory cell induced corrosion has also been reported [57]. This cellular mechanism, although of uncertain biological significance, could also contribute to the total wear particle load. Corrosion metallic particles can be associated with a variable amount of conventional metallic particles generated by adhesion/abrasion through edge loading or neck/ acetabular rim impingement. Their shape varies from round/globular to irregular or rod/needle-like and they usually appear characteristically greenish/yellowish. When observed in large aggregates of nanoparticles or macroparticles of variable size (1 μm to > 500 μm), they can appear as greenish microplates layered with black/reddish streaks (Fig. 4d). Their metal composition varies according to the type of implant and of the wear mechanism [44]. Semithin sections prepared for electron microscopy analysis show nanoparticle aggregates present in the cytoplasm of macrophages in MoM HRA (Fig. 4e), in MoM THA with CoCr MAS with a giant cell containing a large aggregate (Fig. 4f), and Non-MoM THA with CoCr DMN with a large aggregate in inset (Fig. 4g). Corrosion particles can be generated also at metallic interface of fixation device components, as shown in Fig. 4h.

#### Ceramic particles

**Fig. 5:**
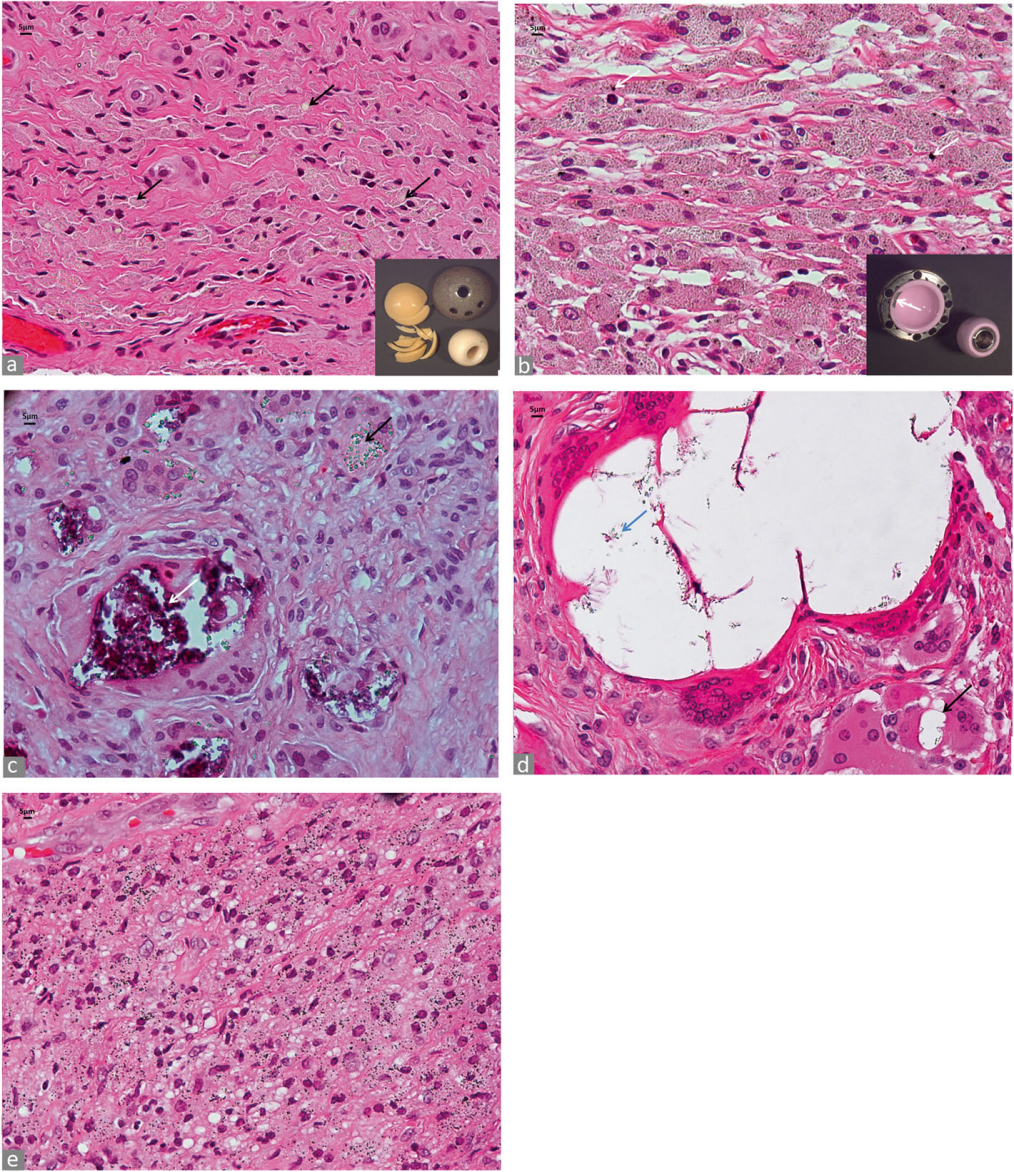
Ceramic/ Polymethyl methacrylate orthopedic cement particles (PMMA). **a** Ceramic particulate debris, small and large microparticles (black arrows) (H&E × 400), fractured alumina ceramic liner in a ceramic-on-ceramic hip implant in inset. **b** Ceramic particulate debris and scattered black particles of conventional metallic debris (white arrows) (H&E × 400); the metallic debris is secondary to neck-to-rim impingement with metal transfer to the ceramic liner (white arrow) in a zirconia toughened alumina ceramic-on-ceramic hip implant with Ti metallic adapter sleeve in inset. **c** Hydroxyapatite. Macrophage/giant cell reaction to deposits of hydroxyapatite (black arrow) with calcification (white arrow) (H&E × 400). **d** PMMA. Large vacuoles of orthopaedic cement dissolved in tissue processing and containing residual particles of radiographic contrast agent (blue arrow) are lined by multinucleated giant cells (H&E × 200) and a smaller vacuole is engulfed by a multinucleated giant cell (black arrow) (H&E × 400). **e** Macrophage infiltrate containing numerous particles of radiographic contrast agent, indicative of third body wear implant failure (H&E × 400)

Ceramics are generally employed in joint replacement arthroplasty as combinations of CoP bearing surface in hip, knee, shoulder, elbow, and ankle implants or CoC bearing surfaces in hip implants. They are classified as: 1) Oxidized ceramics, composed of aluminum oxide ceramic (Al_2_O_3_), zirconium dioxide ceramic (ZrO_2_) or alumina matrix composite (mixed oxide ceramic) with components such as yttrium oxide (Y_2_O_3_), strontium oxide (SrO), and chromium oxide (Cr_2_O_3_); 2) Non-oxide ceramics such as silicon nitride (Si_3_N_4_); 3) Hard coating on metals, such as titanium nitride (TiN); 4) Surface modifications of metals such as a zirconium alloy with 2.5% of niobium through surface oxidation by thermal diffusion; 5) Calcium phosphate ceramics such as hydroxyapatite and tri-calcium phosphate [58, 59]. Wear-induced ceramic particles usually occur in the size range of 20–100 nm and only occasionally up to several micrometers. If only a few particles are present in the macrophages, they are difficult to be identified with certainty and they should be reported only as morphologically compatible with ceramic particulate debris. Abundant ceramic debris is shown in Fig. 5a and b. The larger microparticles observed in Fig. 5a are unusual and probably due to the fracture of the acetabular liner. The birefringence of the microparticles varies from absent to weak, and they exhibit variable shape from globular to irregular-polygonal with sharp edge and colour, from clear to translucent yellowish/greenish/brownish [12, 60, 61] or grey/black (oxidized metal, not shown) according to the type of ceramic of the implant component(s).

#### Hydroxyapatite (HA)

The HA surface coating often used in metal materials (mostly on metallic surfaces) facilitates the osteointegration process of the prosthesis. HA is usually completely replaced by the periprosthetic bone formation and can only be detected by hard grinding techniques in the early phase following implantation and very infrequently as a particulate material in the SLIM. Hydroxyapatite/beta tricalcium phosphates can also be used as bone augmentation agents and are synthesized in sizes ranging from hundreds nanometer to hundreds micrometers [62]. They can present as aggregates of nanoparticles to microparticles with morphological features similar to ceramic particles and can also be associated with calcium deposits and giant cell reaction, as shown in a case treated with hydroxyapatite agent for bone augmentation for massive osteolysis (Fig. 5c).

#### Polymethyl methacrylate orthopaedic cement particles (PMMA)

In the conventional histological tissue preparation, PMMA particles are chemically dissolved during tissue processing. They are identified at light microscopy as empty, multivacuolated cavities of variable size lined or engulfed by multinucleated giant cells (Fig. 5d).

##### Radiographic contrast agent (zirconium dioxide and barium sulfate)

In the vacuoles of the PMMA, which has been dissolved during tissue processing, only the additive, radiological contrast agent zirconium dioxide or barium sulphate is identifiable (Fig. 5d). They are detectable as small, aciniform aggregates of round, slightly birefringent particles with a dark border and a clear center. They can be numerous in the cytoplasm of macrophages, especially in cases of three body-wear and be an indicator of this mode of implant failure (Fig. 5e). Differentiation of zirconium dioxide from barium sulfate is not possible with certainty at light microscopy examination.

### Non-implant related particles

The non-implant related particles present in the periprosthetic soft tissue and/or SLIM include endogenous particles, for the large majority crystals and material related to blood by-products and foreign particles, derived from surgical sutures, fixation/reconstruction devices, joint spacers, skeletal substitute materials, visco-supplements, and drug carriers.

#### Endogenous particles

Endogenous particles are identified by the use of polarized light for the identification of crystals and PBR for the identification of hemosiderin/blood products.

##### Calcium pyrophosphate

**Fig. 6:**
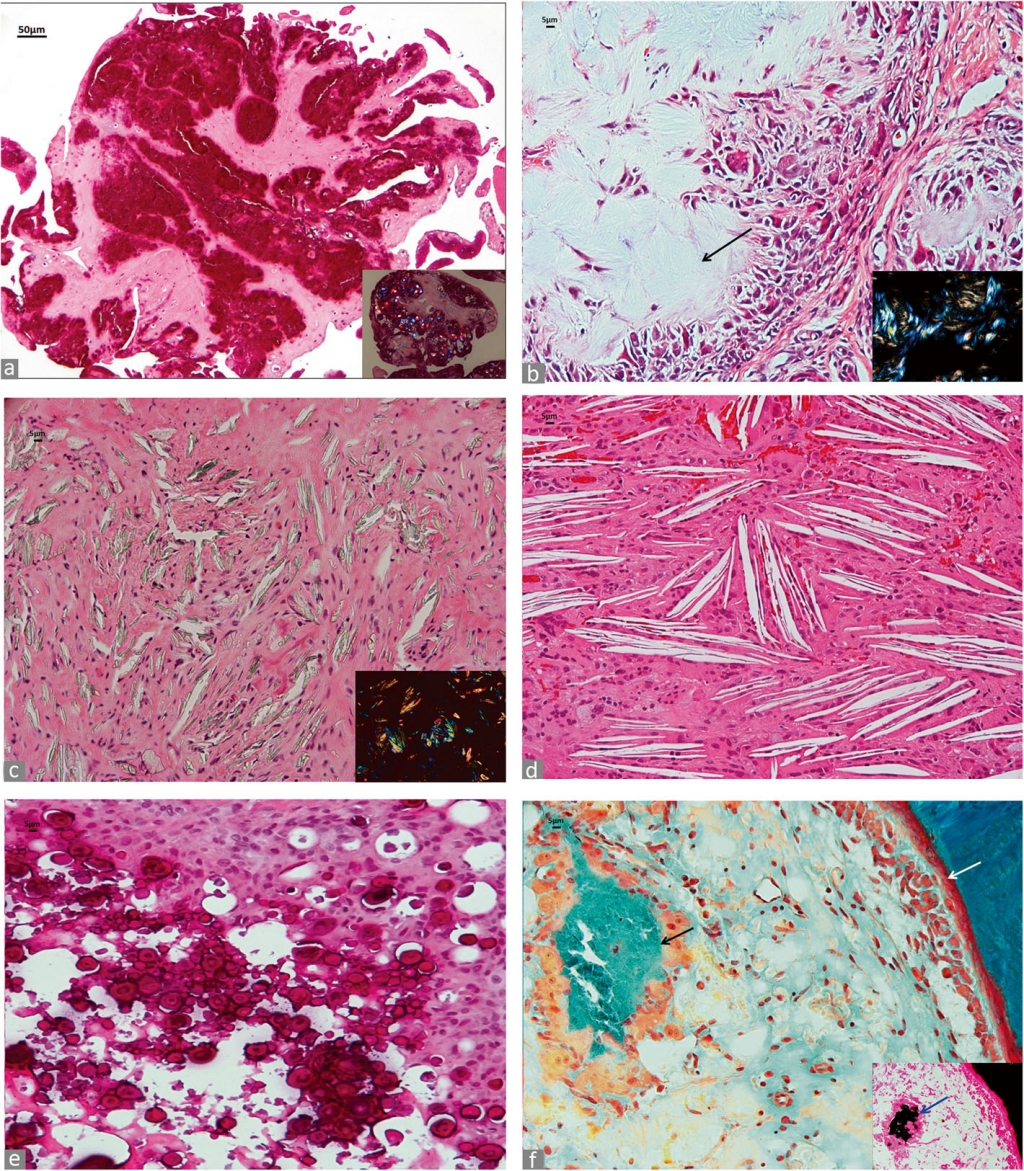
Crystal Deposits. **a** Calcium pyrophosphate (CPP). Synovial sclerosis with large amount of CPP (× 100), CPP positive birefringent crystals with rhomboid shape under polarized light, inset (× 200). **b** Monosodium urate. Macrophage and giant cell reaction to fascicles of dissolved urate crystals (black arrow) (H&E × 200), residual negative birefringent crystals under polarized light in inset (× 400). **c** Oxalate. Deposition of oxalate crystals in bone marrow in a case of primary oxaluria (H&E × 200). Positive birefringent crystals under polarized light in inset (× 400). **d** Cholesterol. Clefts of cholesterol crystals in long-standing chronic bursitis with macrophage reaction with numerous foamy forms to particulate wear debris of MoM THA implant (H&E × 200). **e** Hydroxyapatite (calcinosis). Spherical and targetoid deposits of calcium hydroxyapatite in calcific bursitis of shoulder joint (H&E × 200). **f** Hydroxyapatite (calcinosis). Bone marrow stromal reaction to deposits of calcium hydroxyapatite (black arrow) with brisk osteoblatic activity and thick osteoid seam (white arrow) in a femoral head with periarticular tumoral calcinosis in a case of long-standing scleroderma. Undecalcified bone section (Goldner’s Masson Trichrome × 200) with black calcium hydroxyapatite deposits evident in inset (blue arrow) (Von Kossa × 200)

Calcium pyrophosphate (CPP) exists in the form of characteristically rhomboid shaped crystals admixed to cuboid, parallelepiped, and also needle-shaped forms, approximately ≤1 μm to 1 μm in size [26]. CPP deposits exhibit a weak positive birefringence under compensated polarized light (pale yellow with the long axis perpendicular to the compensator and pale blue when parallel) and are characteristically embedded into a reddish, homogeneous matrix (Fig. 6a). In the periprosthetic soft tissue and/or the SLIM, deposits of calcium pyrophosphate crystals can also be detected in proximity to the macrophage/giant cell reaction to wear debris.

##### Urate

Sodium urate crystals are present in the H&E section in the form of haphazardly arranged short fascicles of needle-shaped, empty spaces corresponding to dissolved urate crystals, embedded into an amorphous, greyish matrix and surrounded by a macrophage/giant cell reaction (Fig. 6b). Since urate crystals are water-soluble, negatively birefringent urate crystals (bright blue with the long axis perpendicular to the compensator and bright yellow when parallel) may only be detected directly in the native preparation or in histological section before paraffin removal [26], although some residual crystal fascicles can be present after staining in large tophi. The particle size can range between 5 and 25 μm.

##### Oxalate

Deposits of calcium oxalate in bone and other tissues is known as oxalosis and it is a secondary to the occurrence of primary hyperoxaluria (PH) due to an autosomal recessive hereditary disorder of the metabolism of glyoxylate, most frequently caused by a enzyme deficit of alanine-glyoxylate aminotransferase (PH type I) located in the hepatic peroxisomes which causes excessive oxalate production with involvement of the kidney, the excretory organ [63]. The crystals appear as pale green or pale yellow arranged in clusters of broken plates or radial rosettes embedded in a fibrous stroma and are birefringent under polarized light (Fig. 6c).

##### Cholesterol

Cholesterol crystals are dissolved during tissue processing and typically appear as haphazardly arranged small fascicles of empty clefts (Fig. 6d). The formation of the crystals in the periprosthetic neo-synovium occurs in arthroplasty after a relative long time of implantation and often in long-standing chronic bursitis with a marked particle-laden macrophage infiltrate with abundant necrotic cell debris.

##### Hydroxyapatite (calcinosis)

Soft tissue deposition of hydroxyapatite can occur in a single or multiple locations and can be related to a number of systemic disorders such as familial or idiopathic tumoral calcinosis, associated with autoimmune rheumatologic disorders and in particular scleroderma, and metabolic conditions such as renal failure with dialysis, hypervitaminosis D, and other disorders of the calcium/phosphorus homeostasis. Although almost any joint can be affected by calcinosis, the shoulder is the most commonly involved region where calcific tendinitis and/or bursitis can occur [64]. The crystals appear spherical and targetoid and are lined by a macrophage/giant cell reaction (Fig. 6e). Deposits with stromal reaction in the bone marrow associated with brisk osteoblastic activity and thick osteoid seam can also be observed in cases of periarticular tumoral calcinosis associated with scleroderma (Fig. 6f).

##### Hemosiderin

**Fig. 7:**
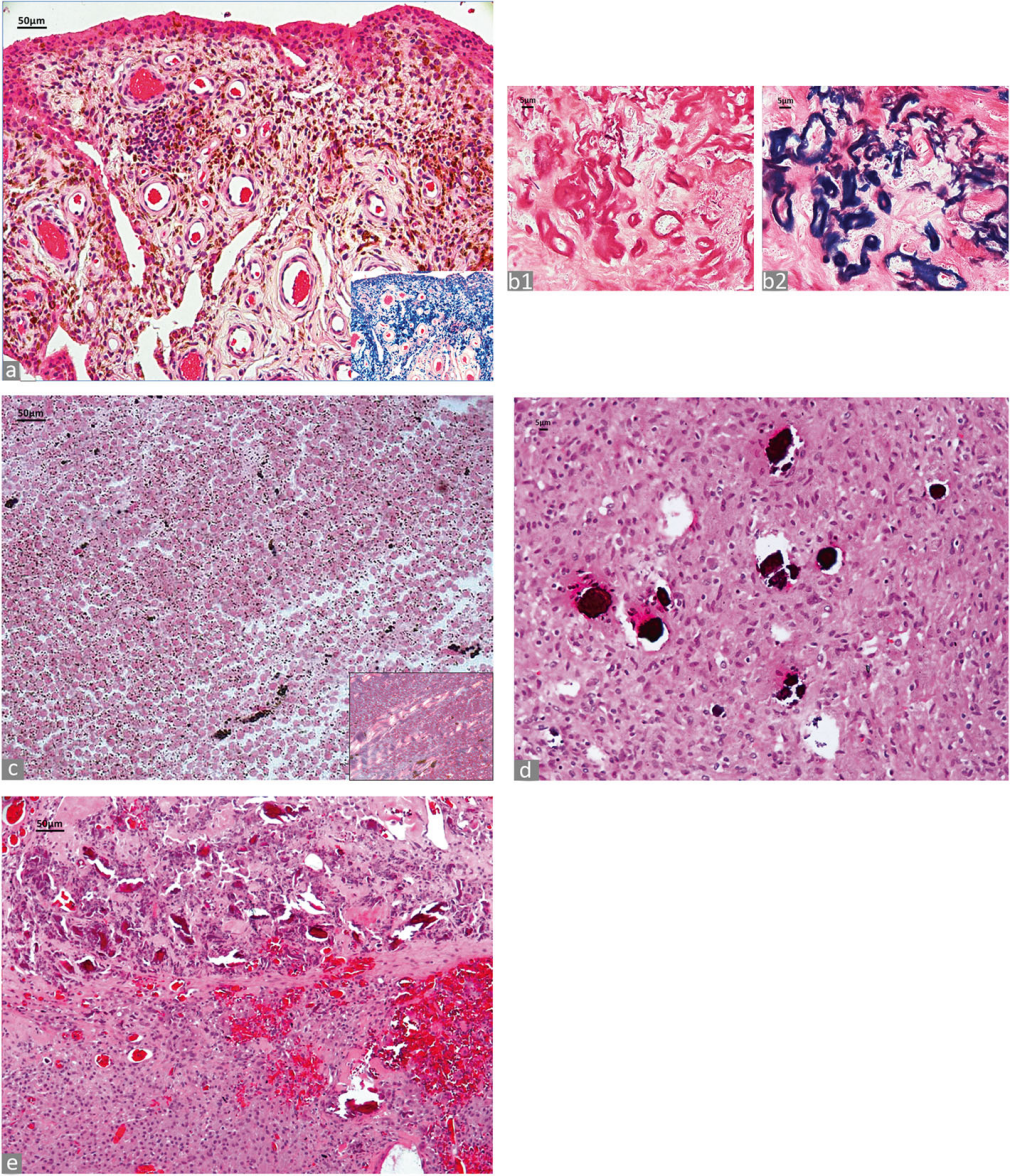
Hemorrhage byproducts and calcific/osseous deposits. **a** Hemosiderin pigment in neo-synovium (H&E × 100), hemosiderin deposits positive for PBR in inset (PBR × 100). **b1** Gamna-Gandy bodies in failed MoP THA with hemorrhage secondary to multiple dislocations (× 400), **b2 **positivity to PBR reaction, PBR stain in inset (× 400). **c** Formalin pigment artifact, negative for PBR reaction (PBR stain × 100) and with typical birefringence of the granules of formalin pigment under polarized light in inset (× 200). **d** Lime (dystrophic calcification) in periprosthetic neo-synovium of a case of hip implant failure (H&E × 200). **e** Neo-synovium of a failed MoP THA implant for aseptic loosening/osteolysis with abundant osseous detritus embedded in the superficial layer (H&E × 100)

Hemosiderin deposits, usually secondary to chronic bleeding in the periprosthetic soft tissue or traumatic events such as implant dislocation or periprosthetic fracture, appear as course, granular golden brown deposits in the macrophage cytoplasm (Fig. 7a). In the Prussian blue reaction, hemosiderin deposits stain intensely dark blue (Fig. 7a, inset). Hemosiderin deposits in macrophages of the SLIM may also be present in conjunction with prosthesis material wear particles and might be difficult to differentiate without the PBR stain from ceramic and/or metallic corrosion particles especially if they have a granular size.

##### Gamna-Gandy bodies

Gamna-Gandy (G-G) bodies are defined as small, spheroidal or irregular yellow-brown foci, consisting of dense fibrous tissue and collagenous fibers encrusted with iron pigments and calcium salts (Fig. 7b1 and b2). They were first described in the spleen early in the twentieth century and were erroneously considered to be caused by fungal infection. Now G-G bodies are considered to result from organization of small hemorrhages and have been characterized by scanning electron microscopy and x-ray fluorescence spectroscopy with demonstration of their crystalline nature and chemical structure of CaPO_4_·FeOH [65]. G-G bodies are a well-recognized finding in atrial myxomas where they form linear arrays of mineral-encrusted fibers, often at the edge of resolving hemorrhages. Their definition has been expanded to include all the formations of connective tissue fibers admixed to iron pigments and calcium salts, irrespective of size or form.

##### Formalin pigment

This pigment is produced by acid acting upon hemoglobin and is also known as acid hematin. The appearance is black to brown with amorphous to microcrystalline granules (Fig. 7c). Formalin pigment granules can be present in the H&E stained histological sections of periprosthetic tissues fixed in formalin having a low pH [66].

##### Lime (calcium carbonate)

Calcium carbonate, as one of the most important forms of lime, appears in the form of basophilic, non-birefringent coagulative deposits (Fig. 7d). Calcium carbonate is observed mostly embedded in a fiber-rich connective tissue with scant macrophage/multinucleated giant cell reaction and can be a consequence of an inflammatory process and/or tissue necrosis. The particle size is usually larger than 1 mm.

##### Bone tissue detritus

The bone tissue fragments are often surrounded by macrophages and osteoclast-like giant cells, particularly in detritus synovitis as a consequence of osteoarthritis, necrosis, and especially rapidly progressive osteoarthritis. In SLIM, the cause can be bone fragmentation secondary to osteolysis with or without fracture and also bone milling during surgery, the so-called cutting-grinding effect, as a by-product of the surgical operation. The particle size usually ranges from 5 to > 300 μm (Fig. 7e).

#### Foreign particles

Foreign particles are predominantly generated by fixation/reconstruction devices and by materials or substances used as fillers or carriers to alleviate the symptoms or complications of joint arthritis and arthrosis.

##### Surgical sutures (absorbable and non-absorbable)

**Fig. 8:**
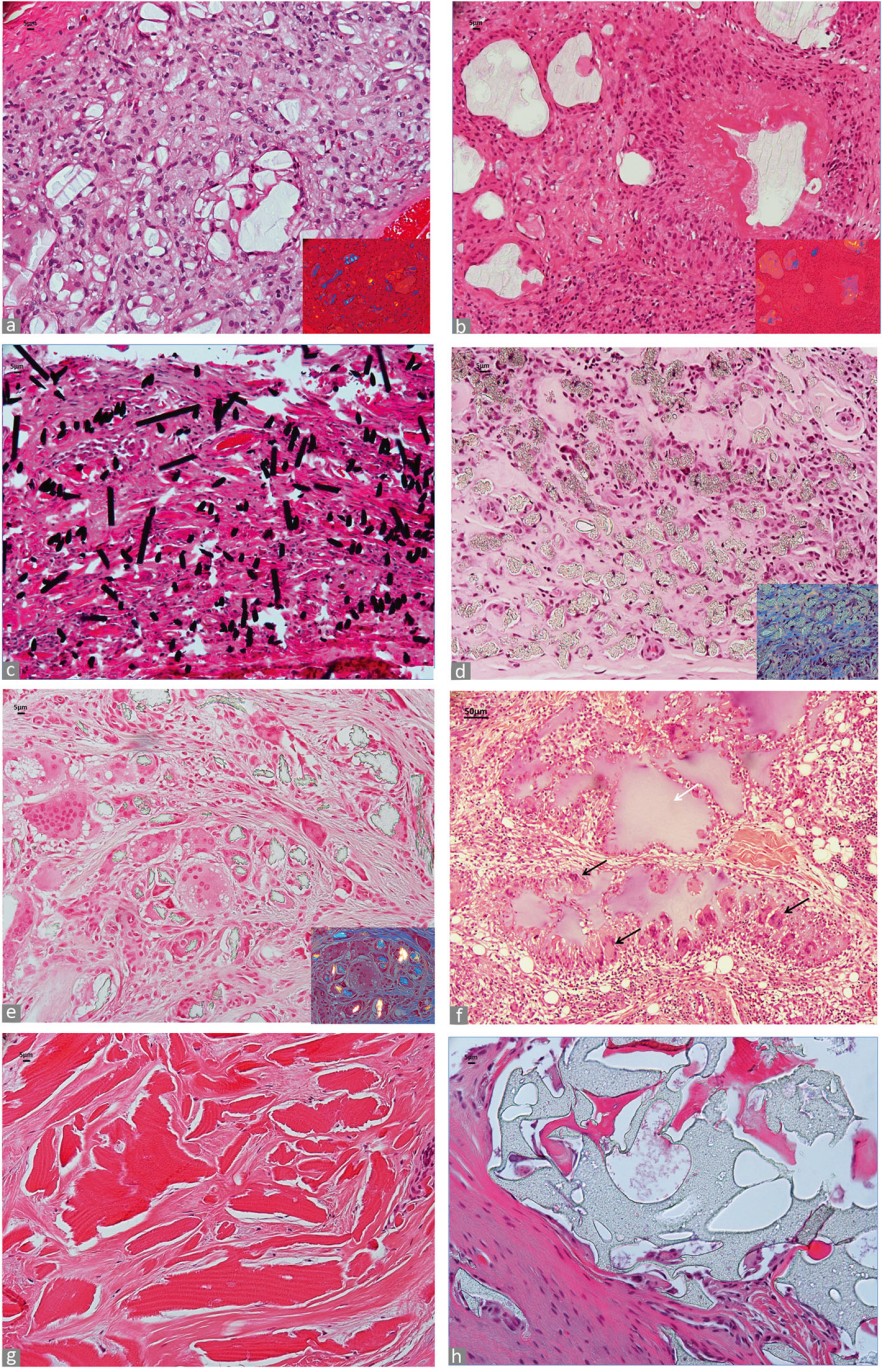
Non-implant wear, foreign particles/bone graft. **a** Absorbable surgical suture. Macrophage/giant cell reaction to deposits of absorbable suture material (H&E × 200), birefringent suture under polarized light in inset (× 200). **b** Fixation device (interference plastic poly-DL-lactide screw). Macrophage/giant cell reaction with palisading macrophages to plastic screw material implanted for anterior cruciate ligament reconstruction (H&E × 200), focal birefringence under polarized light in inset (× 200). **c** Scaffold composite material (carbon and polyester). Reactive fibrous tissue with embedded fragments of carbon fibers from a scaffold class anterior cruciate ligament (H&E × 200). **d** Joint spacer material. Macrophage reaction to silicone elastomer particles from a finger prosthetic implant (H&E × 200); particles are non-reactive under polarized light in inset (× 400). **e** Joint spacer material. Florid giant cell reaction to particles of porous polyurethaneurea (H&E × 200); particles are birefringent under polarized light in inset (× 400). **f** Visco-supplement reaction. Palisading macrophages and giant cell (black arrows) reaction to hyaluronan deposits (white arrow) (H&E × 100). **g** Skeletal substitute material. Demineralized bone matrix of allograft implant with intervening fibrous tissue (H&E × 200). **h** Skeletal substitute material. Porous tricalcium phosphate bone substitute (H&E × 200)

Surgical sutures are usually easy to identify because of their high birefringence and tubular structure in longitudinal, oblique, and cross section or even filamentous structure. Absorbable surgical sutures, however, can be more challenging especially when broken in small fragments because of their heterogeneous birefringence and appearance (Fig. 8a).

##### Fixation/reconstruction devices/materials

Fixation devices which can break down and can represent a differential diagnosis with supra-macroparticulate PE are fixation plastic screws and/or anchors and in our experience birefringence is usually less intense and homogeneous for fixation devices than for large PE particles (Fig. 8b).

Debris released by broken metallic devices, such as metallic plates or acetabular screws cannot be distinguished from metallic wear debris and a clinical history and examination of the explanted hardware is essential for the correct histological diagnosis.

Among the reconstruction materials, an interesting example is represented by the Active Biosynthetic Composite Ligament (ABC), introduced as a scaffold class ligament in 1985 for primary reconstruction of the human anterior cruciate ligament, composed of interwoven carbon and polyester unit material. The artificial ligament failure usually occurs because of stretching and breaking of the fibers secondary to mechanical or fatigue factors [67]. At histological examination carbon fibers appear jet black with a cylinder-like shape and do not polarize. The diameter is approximately 5–8 μm and the length of the fibers is variable; in the non-fragmented state, it can be up to several mm. They are embedded in a fibrous matrix, without presence of a macrophage response (Fig. 8c). A distinct granulomatous foreign body reaction has been described in a single case report [68]; however, examination of the histological picture provided shows only fibrous reaction around the carbon fibers without cellular response.

##### Joint spacers

Silicone elastomer is the most common joint spacer and has been used for decades as an inert spacer for small and medium-size joints, such as fingers, toes, metatarsal-phalangeal, thumb, wrist and elbow [69]. In the so-called silicone synovitis polycyclic, irregular and rectangular macroparticles up to several mm are present in synovial or capsular location, usually resulting from a fracture of the prosthesis with fragmentation and subsequent development of a foreign body giant cell reaction. These fragments are pale white and can be partially dissolved through tissue processing and histologic section staining. They exhibit a variable degree of birefringence under polarized light (Fig. 8d). Other materials have also been used as spacers causing a similar reaction, such as porous polyurethaneurea [70], shown in Fig. 8e.

##### Injected foreign materials (visco-supplements and drug carriers)

Foreign body-induced cases of synovitis are typically observed following applications of intra-articular substances/drugs. Histologically, the specification of the material (in most cases the drug substrate) is not possible unless a detailed clinical history is available, although certain materials, such as visco-supplements, provide a distinctive foreign body reaction of palisading macrophages admixed to multinucleated giant cells [71] (Fig. 8f).

##### Skeletal substitute materials

Although strictly not part of the particle algorithm, skeletal substitute materials are also used to fill voids around failed joint prostheses and/or increase osteointegration and their recognition at histological examination is important to interpret correctly the pathological findings of the specimen [72]. Among those frequently used are demineralized bone matrix (Fig. 8g) and soluble calcium-based granules (Fig. 8h).

### Differential diagnosis of particle laden macrophages and other macrophage diseases in bone

**Fig. 9:**
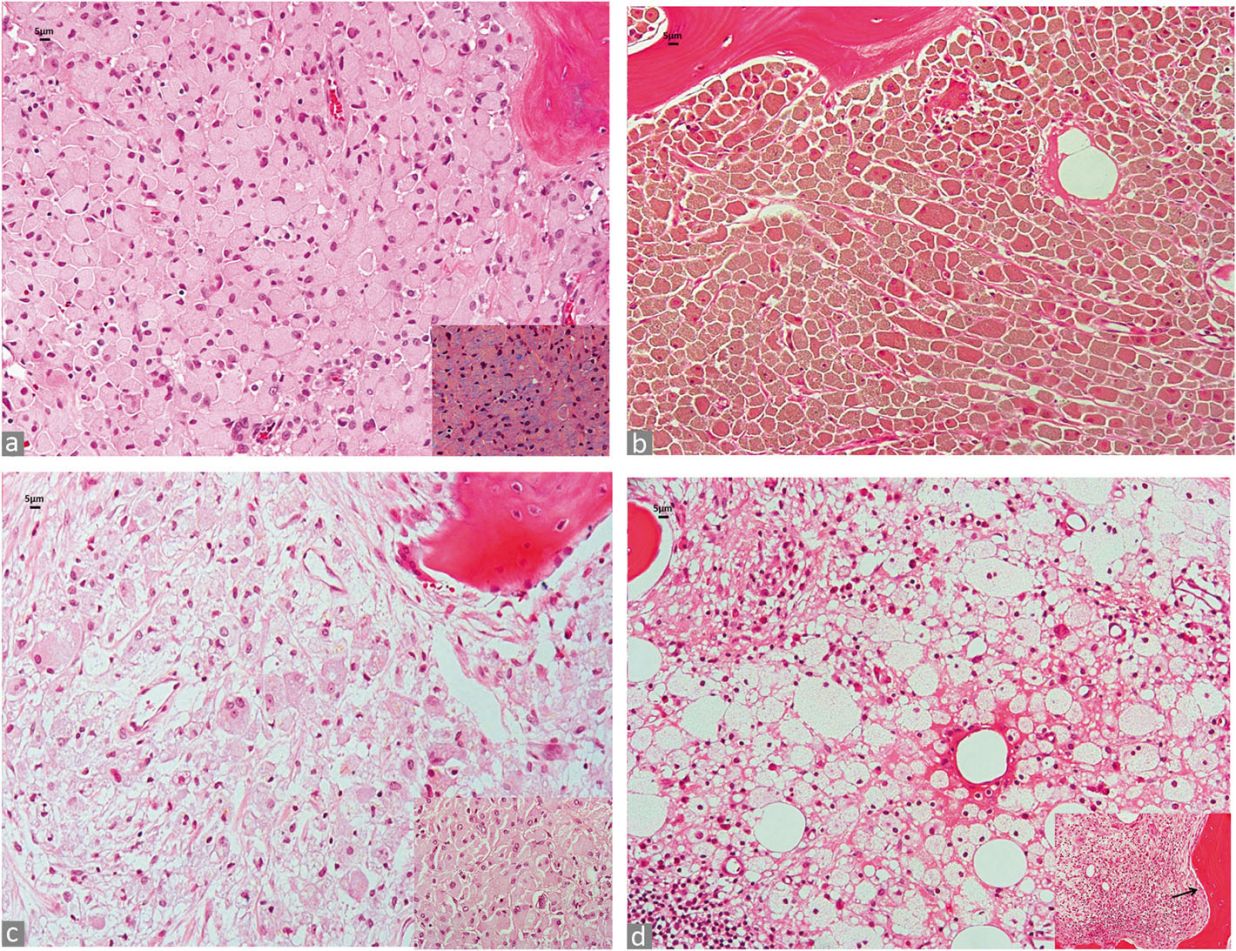
Differential diagnosis between particle laden macrophagic infiltrate and other macrophagic diseases in bone. **a** Implant aseptic loosening (osteolysis). Dense macrophage infiltrate in the bone marrow in a MoP THA implant (H&E × 200) containing birefringent PE microparticles under polarized light in inset (× 400). **b** Implant aseptic loosening (osteolysis). Diffuse, brownish macrophage infiltrate containing tribocorrosion and conventional metallic particles in a femoral head of a MoM HRA implant (H&E × 200). **c** Gaucher’s disease. Macrophage infiltrate in the bone marrow of a femoral head (H&E × 200) with details of the macrophage crumpled cytoplasm in inset (H&E × 400). **d** Erdheim-Chester disease. Bone marrow from proximal tibia with foamy macrophages and mixed chronic inflammatory infiltrate (H&E × 200) and mixed chronic inflammatory infiltrate and adjacent sclerotic cancellous bone with osteoblastic rimming in inset (H&E × 400)

A challenging differential diagnosis can occur in the presence of bone marrow macrophage infiltrates which are characteristic of other conditions, such as lysosomal storage diseases [73] and other macrophage disorders, such as Erdheim-Chester disease (polyostotic sclerosing histiocytosis) [74], especially when limited tissue is available or in consultation practice without a detailed clinical history. Although the diagnosis should be known clinically before the occurrence of a joint prosthetic revision, exceptions can occur because of mild forms of the storage diseases with adulthood onset or misdiagnosis because of their rare occurrence. However, careful examination of the macrophage infiltrate provides clues to differentiate these conditions at light microscopy. The particle laden macrophage infiltrate is usually composed of packed, polygonal macrophages with abundant cytoplasm infiltrating the bone marrow with an easily identifiable particle loading of PE, metal, orthopaedic cement, complex mixed wear; PE particles are seen in Fig. 9a with inset and conventional and tribocorrosion metallic particles are seen in Fig. 9b. The macrophages in the lysosomal storage diseases contain the substance which cannot be digested because of the enzymatic defect, such as in Gaucher’s disease, in which they show characteristic crumpled tissue paper-like cytoplasm (Fig. 9c). In polyostotic sclerosing histiocytosis, the macrophages are lipid laden with abundant foamy cytoplasm and admixed to an inflammatory infiltrate predominantly composed of lymphocytes and plasma cells (Fig. 9d) and if present, the cancellous bone is sclerotic with evident osteoblastic rimming (Fig. 9d, inset).

## Discussion

In general, an algorithm is a logical sequence of actions to be performed for solving a diagnostic or therapeutic problem and is widely used in guideline-oriented medical practice. We propose a histological particle algorithm based on particle-defining criteria which provide a guide to implant wear as well as non-implant related endogenous and foreign particle identification in the periprosthetic tissue and SLIM using conventional histology examined at light microscopy with the aid of polarized light and simple histochemical stains when necessary. This simple and comprehensive flow chart aims at providing complementary information to the diagnostic classification of the periprosthetic neo-synovial membrane, bone, and SLIM reaction to implant wear. The characterization of these particles is defined in accordance with a classification based on size and shape, staining characteristics, and properties under polarized light. The particle algorithm is also designed to assist general surgical pathologists, orthopaedic surgeons and the material scientists in the identification of particulate material in the periprosthetic neo-synovial membrane, bone, and SLIM with minimal methodical complexity.

It needs to be emphasized that the particle algorithm constitutes only a guide to identification of implant by-products on a descriptive level by conventional histological examination. Particularly for metallic and ceramic materials but also for the different types of UHMWPE, the definitive material identification, chemical composition and oxidative status is only possible through the use of physical, high-resolution procedures, for example energy dispersive X-ray spectroscopy (EDS) and Fourier transform infrared spectroscopy (FTIR) [53] or synchrotron micro X-ray absorption spectroscopy (XAS) and X-ray absorption near edge structure (XANES) [75]. For detection of low concentration elements in nano-size wear particles, even more sensitive analytical techniques need to be used, such as TEM and TEM-EDS element mapping, SEM, backscatter scanning electron microscopy (BSEM) and BSEM-EDS element mapping examination, X-Ray diffraction spectrometry (XRD) examination and single particle-inductively coupled plasma-mass spectrometry (SP-ICP-MS), as recently reported [43]. The use of analytical nano-technology is also advocated for complex cases in which several revisions have occurred with different bearing coupling and/or use of materials for implant adherence [76]. It needs also to be taken into account that wear particles are tridimensional objects and that for a correct interpretation of their shape and volume, SEM stereoscopy has been shown to provide the most reliable results [77]. The use of these techniques is particularly important for research purposes and also for the determination of different toxicity/immunogenicity of the particles generated by in vitro wear simulation or in vivo for clinical purposes. It is also important to highlight that wear particles generated by new implant configurations can be missed or misdiagnosed at light microscopy examination. One recent, noteworthy example is represented by the metallic nanoparticles generated by MoM implants which were originally described in 2005 as “cytoplasmic pseudo-inclusions which did not resemble wear debris” [78] and therefore were not counted as metallic wear particles or considered as possible cause of the adverse reaction named aseptic lymphocyte-dominated vasculitis-associated lesion (ALVAL). The first study that correctly characterized the cytoplasmic pseudo-inclusions as metallic wear nanoparticles analysed by TEM and SEM was published six years later in 2011 [79] and later confirmed by a larger study including the same implant configuration [43].

The evaluation of the tissue response pattern(s) is also performed in addition to the identification of implant wear and non-implant related particles for a comprehensive pathological report. The correlation between type of particulate wear and inflammatory response can be important for the choice of the most host-compatible materials and tribological couplings [80]. Due to rapidly advancing developments in prosthesis materials and prosthesis design, new types of wear particles and associated inflammatory response patterns can be detected in the periprosthetic tissue and SLIM by histological examination. These response patterns are determined by biomechanical factors (prosthesis design, loading mode, positioning of implant components, and joint fluid waves), particle properties (composition, size, surface, total burden), and host factors (genetic, immunological, protein corona of particles). For the tissue response classification which goes beyond the scopes of this report, we refer to our previous publications which described it in detail [19, 20].

A particle scoring system is advisable to summarize the most important information for the material scientist and the orthopaedic surgeon: 1) Predominant prosthetic particle material; 2) Minor wear/non-wear components; 3) Non-implant related particle material type, if present. Wear particle assessment has been reported with the use of a semi-quantitative scale based on the number of particles/HPF (× 400 or × 500) or particles/macrophage [8, 81], even for small PE and metallic particles below the optic microscope resolution. Since the wear particles from PE components in MoP and CoP bearing surfaces, metallic in MoM and ceramic in CoC bearing surfaces occur mostly in the nano- and submicron size, it appears that only aggregates can be seen at light microscopy with H&E stain and polarized light without the possibility of a reliable count by number. Therefore the use of a practical semi-quantitative scale of five grades is suggested for the evaluation of the wear particle load within the macrophages with slight modifications from the one reported by Natu [13] and adapted from the assessment of iron overload in liver biopsies [82], following the simple principle of the optic microscope magnifications (eyepiece x objective lens): 0 - no identifiable particles at × 400; 1 - particles identifiable at × 400, 2 - particles identifiable at × 200; 3 - particles identifiable at × 100; 4 - particles identifiable at × 40 and × 25. Particle content is assessed in the areas of maximum macrophage density on 10 consecutive HPF starting at the lowest magnification. A web-based particle algorithm would be desirable for assuring the constant updating of particle identification associated to the inflammatory response patterns. This tool would be particularly useful to provide information on potential new alternative bearing materials in different stages of pre-clinical examination/use, such as polyethereretherketone which has been recently described in animal/human retrieval studies [83]. The proposed particle algorithm will also need further studies for the assessment of its internal and external validity.

The differences in crystalline deposits in the SLIM, the wide variety of prosthesis materials and the diversity of material combinations and particle pathogenesis mechanisms explain the high level of morphological particle heterogeneity in the periprosthetic tissue/SLIM which makes the process of particle identification for diagnostic purposes challenging, especially when the particle burden is below the resolution power of the optic microscope.

A properly conducted and reported histopathological analysis of peri-implant tissue can provide indispensable information on in vivo performance and modalities of failure of orthopaedic implants [84]. The pathology report, issued primarily for the clinical practice and management of the patient and also in consultation practice for diagnostic and/or medico-legal purposes should include the sections below.

### Macroscopic description

1) Neo-synovial and capsular tissue configuration, consistency, colour, three dimensional measurements, optional weight of the wet specimen, bone tissue sampling if present; 2) Description of the removed implant components if available with specification of manufacturer and type (basic prosthetic alloy, bearing pair, modularity, and component serial number optional when available). Description of basic wear analysis at naked eye or with the use of a dissecting microscope and a digital camera may be added, according to the expertise of the examiner or with the assistance of a biomechanical engineer. However, it needs to be stressed that only basic surface characterization is possible and therefore no definitive conclusions should be drawn on the modality of implant failure with the use of this technique. The terminology used for the implant description should be as precise as possible, using the technical documentation provided by each manufacturer. Consultation of the operative report can also provide confirmatory or additional information on the implant components not removed at surgery. Photographic documentation of the revised components of each implant and of relevant soft tissue specimens can be useful at microscopic examination, for retrospective examination of the cases, and for educational purposes.

### Microscopic description

1) Tissue morphology with presence/absence of tissue necrosis/infarction (thickness measurement) and apoptotic cell necrosis with semi-quantitative assessment (slight, moderate, marked); 2) The description of the wear particulate and non-wear particulate material, according to the criteria previously described and mention of the dominant and secondary implant wear material(s); 3) The description of all cell types present with semi-quantitative analysis and relation to the particle wear, including macrophages, fibroblasts, endothelial cells (flat, tall) and the inflammatory cells of the white series: neutrophils, lymphocytes, plasma cells, eosinophils, mast cells. Immunohistochemical and immunofluorescence studies can provide additional, more specific information when necessary.

### Diagnosis

The diagnosis is centred on the type(s) of periprosthetic tissue/SLIM present according to the classification previously reported [20] with the optional addition of the particulate material(s) as described microscopically and the material of the revised implant component(s). A case comment can be added to highlight a discrepancy with the clinical diagnosis, special features, and indications for a specific clinical follow-up.

The concept of wear particle threshold has been proposed by several groups of investigators for polyethylene wear debris in relation to the occurrence of osteolysis and in particular for total hip replacements, suggested as a practical level of 0.05 mm/y for a 28 mm head size [85], although not universally accepted and with concerns related to its general applicability because of too short follow-up of many studies, inadequate definition of osteolysis, use of plain radiographs only for its determination, and consideration of other associated factors which might be more important than particle volume [86] such as the oxidative state of the particles [35].

Moreover, a systematic review of wear and osteolysis outcomes for first-generation HXLPE could not establish the risk for osteolysis for large diameter ( ≥32 mm) metallic femoral heads or ceramic femoral heads of any size and for TKA because of lack of a sufficient number of studies available [36]. For metallic wear nano-particles it has been reported that they can stimulate a higher inflammatory reaction which can be the result of complex biological factors depending on particle size, shape, composition, surface properties [87] and protein corona coating with lipoproteins [6, 41]. Adverse local tissue reactions recently reported in hip implants of different bearing surfaces could be more dependent on particle composition and aggregation than number and volume, as shown in a study comparing metallic particle generation and inflammatory response in three different configurations, MoM HRA, MoM THA, and Non-MoM THA with CoCr dual modular neck [43] and also development of osteolysis dependent on different cytokines according to particle composition and size and macrophage response [87]. Attempt to establish a threshold concentration of Co and Cr blood metal ion has been proposed and recently modified in an attempt to identify patients at risk for adverse tissue reaction progression [88]. To the best of our knowledge, no particle threshold value for osteolysis or other adverse tissue reactions has been reported for ceramic or polymethyl methacrylate orthopedic cement particulate debris. A final word of caution has to be spent for potential, long-term health effects of wear particles and in particular of metallic nano-particulate debris in distant tissues and in contact with bone marrow residing mesenchymal stromal cells [89] and hematopoietic stem cells.

## Conclusions

Due to the continuous developments of new materials and combinations in orthopaedic prostheses, we believe that a web-based particle algorithm would be the ideal set up to assure the constant updating of the materials used for accurate particle identification in the periprosthetic tissue/SLIM.

The histological particle algorithm for detection and identification of implant wear and non-implant related particulate materials in joint arthroplasty should be considered a standard for the histological analysis. It provides a basic, useful tool for particle identification at routine light microscopy examination and it is time-saving and low-cost.

The algorithm can also be used to reduce intra-observer and inter-observer variability in order to provide a common platform for multicentric implant retrieval/radiological/histological studies and valuable data for the risk assessment of implant performance to regional and national implant registries and government agencies.

## Abbreviations

ALTR: Adverse local tissue reaction
ALVAL: Aseptic lymphocytic vasculitis associated lesion
ARMD: Adverse reaction to metallic debris
BSEM: Backscatter scanning electron microscopy
CoC: Ceramic-on-ceramic
CoCr: Cobalt-chromium
CoP: Ceramic-on-plastic
CPLM: Compensated polarized light microscopy
CPP: Calcium pyrophosphate
DMN: Dual modular neck
EDS: Energy-dispersive X-ray spectroscopy
FITR: Fourier infrared transmission spectroscopy
H&E: Hematoxylin and eosin
HPA: Histological particle algorithm
HRA: Hip resurfacing arthroplasty
HXLPE: Highly crossed-linked polyethylene
LHTHA: Large head total hip arthroplasty
MAS: Metallic adapter sleeve
MoP: Metal-on-plastic
Non-MoM DMNTHA: Non-metal-on-metal hip dual modular neck total hip arthroplasty
PH: Primary oxalosis
PMMA: Polymethyl methacrylate orthopaedic cement
SDD: Silicon drift detector
SEM: Scanning electron microscopy
SLIM: Synovial-like interface membrane
SP-ICP-MS: Single particle-inductively coupled plasma–mass spectrometry
TEM: Transmission electron microscopy
THA: Total hip arthroplasty
TKA: Total knee arthroplasty
UHMWPE: Ultra-high molecular weight polyethylene
XANES: X-ray absorption near edge structure
XAS: Synchrotron micro X-ray absorption spectroscopy
XRD: X-ray diffraction spectrometry
